# Understanding the Factors Involved in the Development and Early Implementation of "Pharmacy First" Services for the Management of Common Conditions in England

**DOI:** 10.34172/ijhpm.9442

**Published:** 2026-05-24

**Authors:** Mirza Lalani, Isaac Chu, Agata Pacho, Ayodeji Matuluko, Basharat Hussain, Claire Anderson, Nicholas Mays, Rebecca E. Glover

**Affiliations:** ^1^Department of Health Services Research and Policy, London School of Hygiene & Tropical Medicine, London, UK.; ^2^School of Medicine, University of Nottingham, Nottingham, UK.; ^3^School of Pharmacy, University of Nottingham, Nottingham, UK.; ^4^Department of Public Health, Environments and Society, London School of Hygiene & Tropical Medicine, London, UK.

**Keywords:** Policy Implementation, Community Pharmacy, Primary Care, Qualitative Research, England

## Abstract

**Background:**

Amidst growing pressures on primary care services in England, the Pharmacy First (PF) scheme was introduced in 2024 to enable community pharmacists (CPs) to manage seven common conditions, including supplying antibiotics, where appropriate, according to patient group directions (PGDs). PF aims to increase timely access to care, reduce general practitioner (GP) workloads, and address health inequalities in terms of access to primary healthcare. This paper, part of a wider evaluation of PF, aims to describe and explain the factors affecting its development and early implementation.

**Methods:**

Semi-structured (n=31) qualitative interviews were conducted with policy-makers, representatives of national community pharmacy and general practice bodies and frontline CPs and GPs in England. Analysis was guided by a framework combining Walt and Gilson’s "Policy Triangle" and the Consolidated Framework for Implementation Research (CFIR).

**Results:**

The study identified a range of factors shaping PF development and implementation. These included policy design complexity, stakeholder engagement, political priorities, and contextual pressures such as funding constraints and workforce shortages. Pharmacists welcomed the clinical upskilling opportunity, while GPs voiced concerns about patient safety and duplication of work. A lack of public awareness, inadequate training access (particularly for independent pharmacies and locum CPs), and poor interoperability between community pharmacy and general practice information systems further hindered rollout. Policy "layering," with limited consideration of the implications for existing community pharmacy clinical services and the absence of a phased implementation strategy caused confusion among GPs and patients.

**Conclusion:**

PF illustrates both the potential and challenges of expanding clinical roles in community pharmacy through national policy. Despite political backing and sector-wide engagement, its implementation faced structural, financial, and communication barriers. Realising its full potential requires workforce and integrated information infrastructure, sustainable funding, and clear inter-professional communication and information sharing between general practice and community pharmacy

## Background

**Key Messages**

**Implications for policy makers**

- *Strengthen communication and engagement with new policies like Pharmacy First (PF)*: Provide clear, tailored communication to relevant stakeholders, in this case, GPs, their staff, and the public to raise awareness of PF, clarify its scope, and explain how it integrates with other pharmacy services.
- *Ensure robust information infrastructure*: Improve the interoperability of information systems between community pharmacy and general practice to reduce administrative burden and support seamless patient care.
- *Promote effective models of collaboration between community pharmacy and general practice*: Identify and replicate successful examples of collaboration to enhance PF delivery, uptake, and patient outcomes.
- *Conduct workforce impact analysis*: Incorporate workforce planning into policy design, ensuring strategies are in place to expand resource capacity and support pharmacists to deliver PF and similar clinical services.

**Implications for the public**

Pharmacy First (PF) was introduced to improve access to care by allowing people to receive advice and treatment for common conditions directly from their community pharmacy. Our study found that pharmacists welcomed this expansion of their role, but the rapid rollout of the service created difficulties. These included offering PF despite; staff shortages, less time for training and inadequate information technology (IT) systems that made sharing patient records with general practitioners (GPs) more challenging. Some GPs were cautious about patient safety and the extra workload PF might create. For the public, this means PF may not yet be delivering its full potential. However, if supported with greater awareness among the public, better IT systems, and closer general practice-pharmacy collaboration and communication, PF could reduce pressure on general practice and make it easier for people to get timely care in their communities.

In England, patients access general medical services through general practice and usually require an appointment to see a general practitioner (GP) or other healthcare professional, in contrast to community pharmacies, which provide walk-in access. However a, lack of timely access to general practice has become a pervasive problem with 24% of patients waiting at least a week to see a GP in 2023.^1^ In response, as part of the Government’s National Health Service (NHS) Primary Care Recovery Plan, NHS England (an arm’s-length body of the Department of Health and Social Care) developed and commissioned Pharmacy First (PF).^2^ Since February 2024, community pharmacies participating in PF can supply prescription-only antimicrobials for seven common conditions (See Table 1): acute otitis media, uncomplicated urinary tract infections (UTIs) in women, sore throat, sinusitis, impetigo, shingles, and infected insect bites, after consultation with a community pharmacist (CP)^3^ through patient group directions (PGDs) (written instructions that allow registered healthcare professionals to supply or administer medicines to patients, usually in planned circumstances).

**Table 1:** Key Details of the Pharmacy First Service (England)

**Service Component**	**Description**
Year of national launch	2024
Eligibility	People residing in England/UK
Composition	Seven clinical conditions/pathways
Conditions covered in PF clinical pathways	1. Acute otitis media in children (aged 1 to 17) 2. Uncomplicated UTIs in women aged 16 to 64 3. Sore throat (≥5 years old) 4. Sinusitis (≥12 years old) 5. Impetigo (≥1 year old) 6. Shingles (≥18 years old) 7. Infected insect bites (≥1 year old)
Remuneration details	*February 2024–April 2025*: £15 per PF consultation (assuming certain clinical criteria are met). Documented referral from GP or NHS 111 results in payment, irrespective of the outcome of the consultation £1000 payment for community pharmacy reaching 'threshold' per calendar month – defined as the minimum number of completed PF to be eligible for the payment. The threshold number increased stepwise, from 5 to 30 consultations in the first 14 months of PF service *April 2025 onwards*: £17 per PF consultation *June 2025 onwards*: Fixed payment of £500 for community pharmacies delivering 20 to 29 clinical pathway consultations per calendar month. £1000 maximum total payment for those who deliver 30 or more clinical pathway consultations per calendar month.

Abbreviations: UTI, urinary tract infection; PF, Pharmacy First; GP, general practitioner; NHS, National Health Service.

 In England, PGDs have allowed community pharmacies to manage common ailments such as head lice, threadworm, conjunctivitis, insect bites, and cystitis for the last 20 years.^4^ PF is the latest in a series of nationally commissioned community pharmacy services, building on the NHS Community Pharmacist Consultation Service (CPCS), which allows GPs to refer patients to pharmacies for minor illness advice and treatment.^5^ Furthermore, in 2019, community pharmacies adopted the Community Pharmacy Contractual Framework to provide specific clinical services including an oral contraception service; blood pressure checks; medications management for newly prescribed medications for both long-term conditions and upon discharge from hospital as well as influenza vaccinations.^6^

 PF reflects a wider shift across high income countries toward antimicrobial prescribing by healthcare professionals and serves as a potentially valuable model for countries initiating or expanding similar community pharmacy schemes. For example, in some Canadian and Australian provinces, CPs can prescribe antibiotics for women with uncomplicated UTI.^7,8^ In the UK, both Scotland and Wales have implemented community pharmacy schemes that allow the supply of antibiotics for common conditions, similar to those included under England’s PF. These schemes predate the English service but research on the implementation and impact of the Scottish and Welsh schemes remains limited.^9,10^ Despite the growing interest in expanding clinical services (including antibiotic prescribing) in community pharmacy, there is limited evidence of the key factors that affect implementation of such services.

 The policy ambition for PF in England is significant. As potential PF users can walk into pharmacies to access care, NHS England contended, that PF could free up 10 million GP appointments while also reducing health inequalities, given the higher concentration of pharmacies in deprived areas.^11^ As such, PF serves as a valuable case study for examining how political objectives, contextual pressures, and the roles of key actors can shape both policy development and implementation. These aspects are core dimensions of Walt and Gilson’s Policy Triangle,^12^ one of two frameworks used in this analysis. The second framework, the Consolidated Framework for Implementation Research (CFIR)^13,14^ provides a systematic exploration of the organisational factors that influence how the PF policy is enacted. This study aims to describe and explain the factors affecting policy development and early implementation of PF in England.

## Methods

###  Theoretical Framework

 To cover all policy phases^15^ we used the Policy Triangle and CFIR as complementary frameworks. The Policy Triangle is a conceptual framework used in health policy analysis that aims to capture the key components that influence health policy decisions and outcomes. It highlights how a policy’s content (policy objectives), context (political, social, economic, organisational factors), actors (interest groups), and processes interact in the policy process. The CFIR is a meta-theoretical framework originally comprising 39 constructs arranged across five domains (outer setting, inner setting, individual characteristics, intervention characteristics, and implementation process). It is a practical guide for systematically assessing factors that affect implementation outcomes at different levels (eg, national/local). It has been used to assess the implementation of a range of new policy and practice interventions in health services, including in community pharmacy.^16,17^ The CFIR highlights how a policy may vary in its impacts depending on context and on how it is implemented in different places, prompting the researcher to identify the factors that bring this about. For the purposes of this work we have used the latest version of the CFIR (2022),^14^ while also retaining selected constructs from the original 2009 framework^13^ that were better suited to this type of policy evaluation. Policy development and implementation are often viewed separately but are in fact continuous and dynamic because of feedback loops in most policy systems. This study addresses this separation by combining frameworks for policy analysis and implementation science.

###  Study Setting 

 This research is part of a larger mixed methods evaluation of the implementation of PF.^18^ Qualitative semi-structured interviews were undertaken with purposively sampled key stakeholders in national PF policy, PF programme implementation and PF service delivery roles across England between July-December 2024, during the early implementation phase of PF (4-10 months after its launch).

###  Data Collection

 Semi-structured interviews (n = 31) were held with policy-makers (n = 8), national sector representatives for community pharmacy and general practice (n = 13), and front-line CPs and GPs (n = 11) (See Table 2). Interviewees were identified purposively to ensure participation from the key groups representing community pharmacy and general practice nationally. These included professional bodies involved in negotiating new services in community pharmacy as well as general practice since PF is intended to relieve some of the demand on GPs. Table 2 provides an overview of organisations from which interviewees were selected.All interviews were undertaken using a topic guide which was developed using the integrated framework – the factors that affect the development and implementation of the PF service. The guide was also designed to be adaptable to incorporate further or different questions for subsequent interviews.

**Table 2:** Overview of Interviewees

**Stakeholder Group **	**Organisation/Professional Group**	**Description/Role of Organisation **
Policy-maker (n = 8)	Department of Health and Social Care (n = 2)	Responsible for health and social care policy in England including PF policy development.
	NHS England (n = 6)	Arm’s-length body responsible for PF policy development and implementation.
Sector leader and other national stakeholders (n = 12)	General Pharmaceutical Council	Professional regulator for pharmacists in the UK.
	Royal Pharmaceutical Society	Professional leadership body for pharmacists and pharmaceutical scientists in England. Provides strategic direction for community pharmacy including PF.
	Community Pharmacy England	Representative body for all community pharmacy owners in England. Advocating and lobbying on behalf of community pharmacy for PF to government.
	Pharmacists’ Defence Association	Independent trade union that represents and supports employed and locum pharmacists and students.
	National Pharmacy Association	Membership organisation for individual community pharmacies.
	Company Chemist Association	Trade association for large pharmacy operators (‘multiples’) in England.
	Association of Pharmacy Technicians	Professional leadership body for Pharmacy Technicians in the UK.
	Healthwatch	Independent consumer champion for health and social care in England.
	Royal College of General Practitioners (n = 3)	Professional body for GPs in the UK.
	British Medical Association	A body that represents, supports and negotiates on behalf doctors and medical students in the UK.
Service delivery – frontline professionals (n = 11)	Community pharmacy multiple	
	Community pharmacy independent chain (n = 3)	
	Community pharmacy independent (n = 3) GPs (n = 3)	
	Community pharmacy clinical lead	Individuals that work in integrated care systems acting as a link between community pharmacy and general practice to support implementation of PF as well as other clinical services.

Abbreviations: PF, Pharmacy First; GPs, general practitioners; NHS, National Health Service.

 The research team comprised senior and post-doctoral researchers with extensive experience in qualitative health services and policy research, including prior research in primary care and community pharmacy, which informed the study design and interpretation of findings. Interviewees were contacted via email. Written informed consent was obtained from all interviewees prior to interview, and all data were handled confidentially. There were no refusals to participate. Interviews lasted 45-60 minutes and were held online via Zoom or Microsoft Teams. One interview was held in person. Interview audio-recordings were then sent for transcription by a specialist company, who anonymised transcripts, removing identifying information, with the resulting data stored securely on password protected institutional servers accessible only to the research team. A COREQ (COnsolidated criteria for REporting Qualitative research) was used to ensure comprehensive, and rigorous reporting of this qualitative study.

###  Data Analysis

 Using a thematic approach, data were analysed deductively (guided by the questions in the interview topic guide) and inductively (where interviewees discussed issues not directly covered in the topic guide).^19^ A coding framework (as per Figure), informed by the Policy Triangle and CFIR (2009, 2022), structured the analysis. NVivo 12 facilitated data management and coding, with the five CFIR/Policy Triangle domains as primary deductive nodes and CFIR constructs and emergent themes as secondary nodes. The inductive themes identified in the data and included in the framework were historical context, social norms and policy or political factors. To enhance credibility and trustworthiness, we held regular team meetings to discuss coding decisions, refine themes, and resolve discrepancies. Approximately 20% of transcripts were double coded to enhance the validity of the analysis. Reflexive discussions were also held throughout to consider how researchers’ professional backgrounds may have shaped data interpretation. The topic guide is provided in Supplementary file 1.

**Figure:**
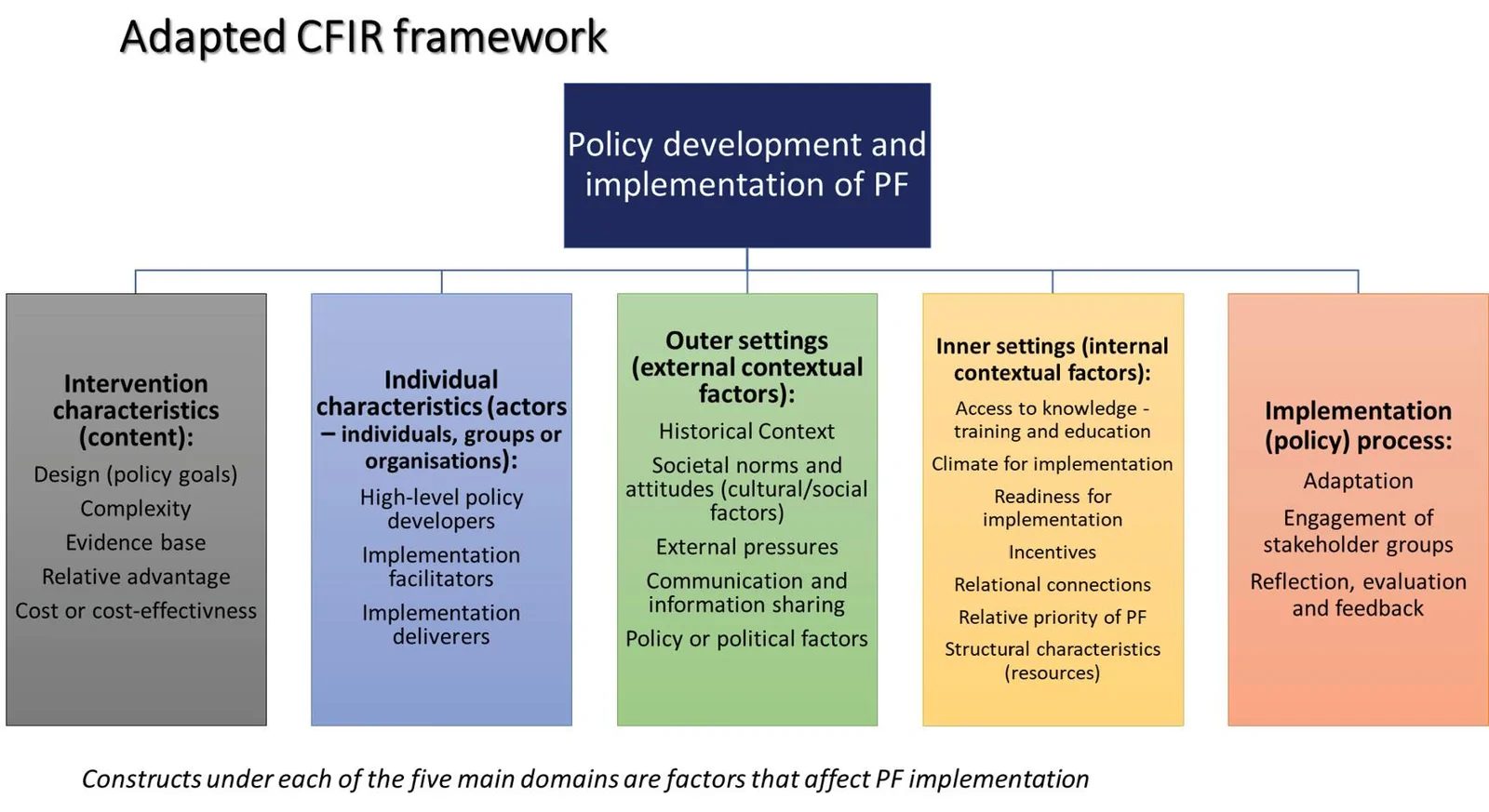
Integrated framework of the Policy Triangle, CFIR (2009) and CFIR (2022). Abbreviations: CFIR, Consolidated Framework for Implementation Research; PF, Pharmacy First.

## Results

 The findings are presented using our integrated framework themes as factors that affect both development and early implementation of PF as a policy and a service.

###  Intervention Characteristics (Policy Content) 

 This domain covers PF policy content, its design, goals, complexity, evidence base, relative advantage, and cost and how these factors influence its development and early implementation.

####  Design (Including Policy Goals) 

 Interviewees identified the key policy goal of PF as increasing access to timely, same-day care to community pharmacy, therefore reducing the burden on general practice. Some interviewees thought PF would promote self-care and shift patient health seeking behaviour toward non-general practice services for acute health problems. Several interviewees also suggested that PF could reduce inequalities in primary care access by addressing unmet need through community pharmacies, while others questioned its capacity to achieve this given the scale of unmet demand. Indeed, PF was considered as means of precipitating change in the case-mix of patients seen by GPs – reducing the volume of patients with minor conditions that visit a GP, although some thought any free capacity created would quickly be filled by patients with more complex needs.

 “*We talk about a huge unmet need in general practice. There will be always an unmet need. The more you provide, the more people will need. You take away a lot. There may be a lot of people that by opening more channels, there will be more of an unmet need. So, you can’t necessarily say that by doing this one thing, you’re going to take away 10 million appointments, because actually, you’re also going to address so many million appointments of unmet need” *(National GP stakeholder).

 Policy-makers and community pharmacy stakeholders felt PF’s clinical pathways would support implementation, as the seven conditions aligned to pharmacists’ skills and routine practice. They also noted these conditions were commonly seen in general practice, hence shifting their management to community pharmacy could help achieve the policy goal of reducing general practice demand.

 Pharmacists and GPs highlighted issues associated with PF referral pathways that could hinder implementation. GPs mentioned that referring to PF may be unnecessary – patients presenting in general practice with symptoms covered by PF pathways triggered internal safety protocols obligating GPs to manage the patient directly. Also, GPs thought PF had the potential to add to their workload, not reduce it; pharmacists were thought to refer patients to general practice unnecessarily. Also, referrals could be made late in the afternoon when appointments were limited, yet GPs were compelled to see the patient. In some areas, community pharmacy clinical leads were working with GPs to create additional appointments at the end of the day to see PF referred patients.

####  Complexity 

 Some interviewees remarked on how complexity had been created by introducing seven conditions at once in England. Pharmacists had to absorb a lot of information about PF at pace with little time for training. PF was introduced alongside pre-existing schemes which treat similar symptoms to those included under PF such as Minor Ailments schemes (which enable access to free treatment for minor conditions directly from a community pharmacy, without seeing a GP) and other locally commissioned services. GPs expressed confusion about which service to refer into as it was not clear how PF differs or links to these other services. Conversely, policy-makers felt an incremental rollout might have reduced impact, leading to lower engagement and public and GP confusion about where conditions could be treated.

 “*I perhaps wouldn’t start it off with ear examinations. Perhaps start off with something that pharmacists would be more comfortable with and then extend out from there. Again, the timeliness to this and people having time to read the volumes of documentation and get themselves ready. If it had started out with just a couple, it might’ve been a better way to have won some hearts and minds” *(CP).

####  Evidence Base

 Sources of evidence reported to have informed the development of PF included learning from similar schemes in Scotland and Wales as well as the experience of locally commissioned services in England. Most interviewees felt that while there were comparable features between the schemes in the three nations, England had largely developed PF independently.

 “*I think England have approached it in their own way. I think that’s just a cultural thing, a political thing. Like to do things in their own way and not talk to each other. So I think that definitely played out. And a number of times that we’ve suggested just lift and shift what they’re doing in Scotland, it will be an awful lot easier, but that didn’t happen for whatever reason” *(National community pharmacy stakeholder).

####  Relative Advantage 

 National community pharmacy stakeholders noted that community pharmacies’ presence in local communities made them well-suited to deliver PF. The convenience to the public of accessing a qualified healthcare professional in a community setting for several minor conditions was perceived to be an appealing feature of PF. However, some policy-makers identified an important drawback of the ease of accessibility of community pharmacy – PF had created thousands of additional access points for antimicrobials and could increase the volume prescribed, thereby increasing the risk of antimicrobial resistance.

 “*But there was a concern at higher levels that patients might end up sort of shopping around and getting multiple antibiotic prescriptions. And effectively we’d end up with a system that almost ended up with antibiotics being available over the counter if it wasn’t monitored effectively”* (Policy-maker).

####  Cost or Cost-Effectiveness

 Many interviewees identified costs as a barrier to PF implementation. Although initial government funding supported its launch, ongoing expenses such as hiring additional staff (eg, second pharmacists), training non-pharmacist pharmacy staff, maintaining equipment (eg, otoscopes), and limited returns on capital investments in constructing new consultation rooms were seen as potential barriers to sustained participation in PF. Some GPs were concerned PF might duplicate work, reducing cost-effectiveness. In contrast, some national community pharmacy stakeholders believed task-shifting to pharmacists could lower the cost per consultation when compared to general practice.

###  Individual Characteristics (Actors) – Individuals, Groups or Organisations Involved in Implementation

 This domain highlights how the roles and characteristics of individuals, groups or organisations involved in PF may affect its development and early implementation.

####  High-Level Policy Developers 

 National organisations such as NHS England, Community Pharmacy England, the Royal Pharmaceutical Society, and the Royal College of General Practitioners contributed to PF policy development, gathering evidence, designing pathways, engaging stakeholders, and lobbying for change. Many interviewees highlighted the then Prime Minister’s key role in elevating PF on the national agenda, linking it to his personal background and incorporating it into the Primary Care Recovery Plan.

 “*Given Rishi Sunak, you know, irrelevant to your political colours, his mum being a pharmacist, you know, I was fortunate enough to go to Number 10 and chat to [name] who was his Spad [special political adviser] for healthcare, and said that Pharmacy First was Rishi Sunak’s primary health transformation policy. It was his go-to health policy. So he was committed personally” *(National community pharmacy stakeholder).

 While this created a strong political impetus, several interviewees viewed the timing of PF’s launch as politically driven, aligning with the UK General Election in mid-2024. Post-COVID-19 pressures including a high demand for GP appointments had already prompted the search for solutions to alleviate pressure on general practice, with PF ultimately emerging as the preferred option.

####  Implementation Facilitators

 At the meso level in sub-regional health and social care systems, known as integrated care systems a new role, the community pharmacy clinical lead had been created to link community pharmacy and general practice – supporting PF and other clinical services. Most clinical leads are CPs and hold dual roles in community pharmacy and general practice. These clinical leads were considered to have facilitated the uptake of PF.

####  Implementation Deliverers

 At the service delivery level, individual pharmacists had developed specific approaches to optimise implementation. Some promoted PF services in their local areas by printing leaflets and posters to display in their pharmacies, delivering talks to local community groups about community pharmacy services (including PF), holding meetings with their local general practice to discuss PF and other clinical services and using social media platforms to advertise PF.

###  Outer Setting

 This section outlines external factors affecting PF’s development and early implementation, including historical context, social/cultural attitudes, external pressures, communication, and policy/political influences.

####  Historical Context 

 Several interviewees remarked how PF was a culmination of the gradual expansion of scope practice of CPs. They pointed to several precursors to PF in England such as Minor Ailments Schemes, emergency contraception pilots, locally commissioned services (eg, for UTI and conjunctivitis) and more recently CPCS. An important milestone was the introduction of a policy in England within the last decade that restricted GPs in prescribing certain medicines/medical products – these are now only available over the counter in community pharmacy and some have been incorporated into Minor Ailment Schemes.

####  Societal Norms and Attitudes (Cultural/Social Factors)

 Community pharmacy sector interviewees noted that the COVID-19 pandemic boosted public engagement with community pharmacy which remained open and accessible throughout. Some mentioned that community pharmacies’ location at the heart of communities meant that patients were more familiar with their local pharmacist than their GP and therefore CPs could support a continuity of care that was diminishing in general practice in the absence of family doctors.

 “*We very rarely talk about continuity of care in relation to pharmacists. And actually I think for certain conditions you know, what we’ve heard from some patients is they like the security and the comfort of seeing the same person every time who knows them, who especially on sensitive issues they feel more comfortable with somebody that they know” *(Policy-maker).

 Community pharmacy interviewees noted shifting public attitudes: community pharmacy was once seen mainly as a place for rapid over-the-counter advice, but PF could drive a paradigm shift, positioning community pharmacy as a source of confidential clinical consultations with a qualified health professional. This would also foster a more patient-centred and less transactional pharmacist-patient relationship.

####  External Pressures

 All interviewees highlighted the challenging context within which PF was introduced in terms ofwider system pressures and pervasive issues in the community pharmacy sector which could impede implementation. Furthermore, longstanding issues in community pharmacy such as a reduction in real terms funding of the core NHS contract (which has led to pharmacy closures), workforce shortages (because of more pharmacists working in general practice) and medicines shortages have made PF an imperative for community pharmacy as its funding may offset cuts from diminishing reimbursement from the core NHS community pharmacy contract.

####  Communication and Information Sharing

 Communication and information sharing about the PF service to the public and general practice was primarily the responsibility of NHS England. At the time of interviews, community pharmacy sector interviewees cited low public awareness of PF as limiting its use. This was despite extensive media coverage and a launch campaign along with a follow up campaign in Autumn 2024. A lack of awareness was compounded by confusion caused by early PF promotional material showing a pharmacist examining an adult ear, despite only children being eligible for the otitis media pathway. Limited awareness of PF among GPs and their staff likely contributed to reportedly variable referral rates and hence, service uptake. Gaps in understanding of GPs and their staff pertaining to PF’s clinical pathway eligibility criteria led to inappropriate referrals.

 “*The restrictions make the whole system much more complicated for people to understand. It’s between this age and this age that you can see a patient….it causes confusion for the GPs and the staff who are referring patients to there, but also for the patients. Because the patient is not really going to know the difference, if they think they’ve got a UTI and they’re 62, or if they’re 72, they’re not going to know the difference between when they can access, when they can’t access a pharmacy service” *(GP).

 A major barrier to implementation was the lack of interoperability between community pharmacy and general practice information systems, despite initial national commitments to enable record sharing soon after PF’s launch. shortly thereafter, GP connect (which allows authorised providers to access GP records) permissions were withdrawn from third parties during GP industrial action, forcing community pharmacies to email PF consultation records. From the GP perspective, only one of the two main patient recording systems included an embedded PF referral form, limiting referrals from practices using the other.

####  Policy and Political Factors

 Policy and political factors shaped stakeholder engagement and implementation. GPs expressed frustration that PF funding was not directed to general practice, particularly amid declining real-term investment. They perceived PF as part of a broader shift of delegation of tasks traditionally performed by doctors to health professionals that may the lack adequate competencies, posing a risk to patient safety. Specifically, GPs worried that pharmacists might misdiagnose conditions, overlook serious illness, or that antibiotics could mask severe disease, with risks compounded if patients consulted multiple community pharmacies without safeguards.

 “*So, if a 58-year-old woman has been to six different pharmacies is that really getting picked up on that she’s got recurring UTIs? And they come to see us and actually it’s lichen sclerosus and actually she needs examining, or is it post-menopausal symptoms really, not anything to do with the UTI? […] So, I think there’s risks, quite significant risks, around this...”* (GP).

 Some GPs suggested that policy-makers should have prioritised expanding the scope of practice of CPs by providing greater flexibility to change specific features of prescriptions, especially in instances of medicine shortages. Pharmacist queries for such issues created additional work for GPs.

###  Inner Setting (Internal Contextual Factors)

 The inner setting refers to internal contextual factors that affect implementation. These factors are pertinent to community pharmacy but also its interaction with the wider primary care sector.

####  Climate for Implementation 

 Both policy-makers and community pharmacy interviewees suggested that there was a growing interest among CPs to adopt an extended scope of clinical practice which would support implementation of PF. This had been accelerated by different local and nationally commissioned schemes prior to PF. Also, before PF, pharmacists often advised on symptoms of the PF conditions and identified when antibiotics might be needed but could only refer patients to a GP which could be problematic during out of hours periods when general practice was closed.

 “*I got to the point where either I had to be a prescriber, or not do community pharmacy … I cannot cope with this because it’s just constantly saying ‘no’ to people. You know what’s wrong with them, they know what’s wrong with them, then you’re sending them to goodness knows where” *(CP).

####  Readiness for Implementation 

 Interviewees remarked on a very short establishment period for PF (three months over the winter of 2023/2024) as a possible barrier to implementation as it exerted considerable pressure on community pharmacies to prepare for roll out. This was reflected in the variation in preparedness across the sector. Community pharmacies that had previously offered locally commissioned services were better prepared to deliver the service due to adequate levels of staffing (eg, an additional pharmacist or accredited checking staff – qualified to check the accuracy of dispensing against a prescription) and training as well as more space within the pharmacy (eg, an additional consultation room).

####  Access to Knowledge – training and Education

 National community pharmacy stakeholders and CPs noted that independent pharmacies (local, small business) had less access to training in comparison to multiples (national or regional companies that own a chain of community pharmacies), leading to variation in early PF implementation. Some multiples provided in-house training to support community pharmacy staff with PF rollout. Training sessions were also organised by NHS England at quite short notice and were oversubscribed. Pharmacists felt that overall training provision (outside the multiples) had been poorly planned with locums especially struggling to access training.

 “*And there were queues in all the training sites … we’ve had loads of feedback from our members, you might get 60 people in a room with three tables. So you’ve got 20 of them crowding round one room to try and figure out how to work an otoscope. You can barely see what’s going on at the table let alone handle it and figure out. You’ve got 10 minutes to be told how to use it and then you’re on the next table because you’re being trained on the next service” *(National community pharmacy stakeholder).

 Training for PF was not mandatory as had been the case with previous locally commissioned services which had required accreditation to provide certain services. This was new territory for pharmacists, and national community pharmacy stakeholders suggested it contributed to some lacking confidence in delivering the service during early implementation.

####  Relational Connections 

 The extent of uptake of the PF service was reported to be mixed across England in part due to reportedly variable general practice referral rates. PF was deemed to be working well where there were pre-existing good relationships with general practice. Higher referral rates were attributed to: (*a*) localities where locally commissioned services existed (GPs more aware of the clinical service provision in a community pharmacy), (*b*) where CPCS engagement was good, and (*c*) where there was geographical proximity of community pharmacies and general practice (eg, health centre pharmacies). In some cases, relationship building was brokered by community pharmacy clinical leads, who were able to use their local knowledge and personal relationships to connect community pharmacy and general practice, as had been the case with CPCS.

 “*we’d already done quite a lot of work with CPCS. We’d tried to launch that to practices (GP) alongside the local community pharmacies. So, we did a meeting where both parties met up and we were able to listen to what the pharmacies were proposing and how the two might work together. We managed to work through numerous barriers, from both sides through a bit of misunderstanding and misinformation a little bit. When Pharmacy First happened, it was a natural transition because we’d already got those conversations going. We’d already got those referral pathways, both forward and backward”* (Community pharmacy clinical lead).

####  Structural Characteristics (Resources)

 Structural characteristics relate to workforce, technological and infrastructure elements that are pertinent to community pharmacy and hence may affect the implementation of PF.

*Workforce: *National community pharmacy stakeholders suggested that pharmacies with adequately skilled staff were better placed to implement PF. Additional staffing released pharmacists to focus on delivering clinical services with less involvement in the dispensing process. Some interviewees suggested that newly implemented legislation that permits pharmacy technicians to deliver PGDs could support pharmacists to focus on PF.

 “*I think infrastructure in terms of staff support, time, cost, when the pharmacist is in doing these consultations, they’re therefore not available to do other things. In very busy pharmacies, we’ve seen that they’ve struggled to be able to do the services and be able to also continue to dispense medications. Some pharmacies have two pharmacists and they have that flexibility but having two pharmacists comes at a significant cost” *(CP).

*Information technology (IT):* A lack of consistent and comprehensive IT provision was seen as a barrier to PF implementation. Issues cited included variable IT infrastructure eg, poor broadband speeds in rural areas, PharmOutcomes (a pharmacy IT software system covering around 90% of community pharmacies in England) introducing multi-factor authentication early in the roll out of PF and the interoperability issues between community pharmacy and general practice outlined in a previous section.

*Physical infrastructure: *A key enabler of PF implementation was the community pharmacy infrastructure, especially effective use of one or more consultation rooms. Construction costs for an additional consultation room were estimated at between £10 000 and £12 000. Pharmacies saw this as a costly investment with concerns about return on investment if threshold targets were not met.

####  Incentives

 National community pharmacy stakeholders and CPs consistently raised issues with meeting monthly threshold payments for PF – funds deemed vital to the community pharmacy sector. Those that met thresholds attributed success to existing community pharmacy Independent Prescriber schemes and good relationships locally with GPs. The clinical criteria that need to be met on PF pathways to trigger payment were thought to be set too high, often resulting in pharmacists spending significant time with patients without remuneration. While seasonality was also thought to account for variable rates of PF consultations. There were concerns among community pharmacy leaders that pharmacies consistently failing to meet threshold targets would struggle to maintain engagement in PF.

 “.*..if you look at the clinical pathways, the gateway (payment threshold) on the most pathways is too high. So you have to meet probably 70% of it or all the criteria to get paid for delivering that service. To get a patient to that gateway point is tough and you have to do a lot of work. There’s probably between four and five patients that you need to see to get one patient through that pathway. The second is there’s a seasonality element to it – if you look at the conditions that are in there, you’ve got some that are more winter based and you’ve got some summer ones”* (CP).

 Pharmacists offered solutions such as (*a*) making threshold payments proportionate to the number of PF consultations and (*b*) thresholds tailored to different community pharmacies in terms of size or dispensing volume. Policy-makers were already considering such changes to PF to increase engagement and uptake.

####  Relative Priority of Pharmacy First

 PF was implemented at a time when there are several competing priorities facing community pharmacies which could act as barriers to implementation. A reduction in the core funding for dispensing medications which has precipitated some community pharmacy closures resulted in some pharmacies embracing PF to close the funding gap. Others mentioned that PF would have to be balanced against the need to deliver other clinical services such as Flu/COVID-19 vaccination. The extent to which PF was prioritised would depend upon pharmacy resources but also the amount of income in relation to the effort required; eg, 4-5 vaccinations can be administered in the time it takes for a single PF consultation.

###  Implementation Process 

 The section outlines how PF implementation has progressed with some reflections on the first 3-10 months of the service.

####  Adaptation

 National community pharmacy stakeholders and CPs described how context-specific adaptation of PF services had taken place amidst the evolving nature of PF implementation. Multiples were considering scheduled appointment slots, while independent pharmacies operated on a first-come, first-served basis, depending on staffing. It was believed that some pharmacies may integrate PF into their routine workflow by delegating tasks to non-pharmacist staff members, hiring additional pharmacists, and providing all staff members with basic knowledge of PF to facilitate service uptake..

 “*…we’re always evolving our approach when people are coming in and seeking medication advice. And we’re doing a lot of free advice most of the time, and they want to buy certain OTC products and things like that. You go to general trading and if we feel that some people can benefit, and fall in any of those seven categories. Then we’ll try to convert those into Pharmacy First if possible, if they meet the gateway criteria”* (CP).

####  Engagement of Stakeholder Groups

 The extent to which different stakeholder groups (pharmacists, pharmacy staff, GPs, and their staff as well as the public) engage with the PF service is a key factor that can affect implementation. Engagement with PF varied across and within integrated care systems exemplified by the bi-directional referral challenges reported earlier. National community pharmacy stakeholders noted only 5% of PF consultations followed GP referrals, though some areas saw higher rates due to strong GP relationships. Limited GP awareness and lack of referral incentives for general practice may explain the variation. Some pharmacists were frustrated by low public uptake, despite efforts at public engagement. They hoped relationships built during COVID-19 vaccination would boost long-term PF use.

####  Reflection, Evaluation and Feedback

 Interviews revealed uncertainty about how national agencies would monitor PF data for improvement, whether evaluation and feedback mechanisms existed beyond the evaluation of which the current analysis is part, and if implementation outcomes were being measured.

 Multiple interviewees described the use of data monitoring to assess implementation progress such as the number of PF consultations and referrals from general practice, while acknowledging limitations in the current approach. The planned collection and analysis of prescribing data were deemed crucial for antimicrobial stewardship concerns. Several respondents described robust monitoring systems to track antibiotic prescribing rates in seven PF conditions, which served both as an implementation quality measure and as reassurance regarding appropriate alignment with antimicrobial stewardship.

 “*We think that the right reporting is in-place. We know what‘s happening. We know how many drugs are going out. We know what‘s going on in the pathways. So I think it is a case of just continuing to monitor that, getting underneath the data and keeping a lens on it, and making sure we build in the understanding from the evaluation” *(Policy-maker).

 Policy-makers valued formal and informal monitoring channels for offering contextual insights, especially into barriers and facilitators of PF implementation that quantitative data could not capture. Mechanisms for collecting patient feedback on PF appeared underdeveloped. While some pharmacies gathered feedback at the point of access, many interviewees saw this as a major gap in evaluating user experience and quality of implementation.

## Discussion

 This study presents stakeholder perspectives on the development and early implementation of PF in England. Senior politicians drove rapid rollout of the service, but the pace of implementation resulted in unequal training access between independent pharmacies and multiples, limited information system interoperability between community pharmacies and general practice, and insufficient capital investment. Service uptake has been constrained by staffing pressures, competing priorities (eg, flu vaccinations), and limited public and GP awareness. Even so, the community pharmacy sector broadly welcomed PF for enhancing pharmacists’ clinical roles. However, GPs were seen as less willing to engage, citing doubts about pharmacists’ competence to provide effective care for the seven PF conditions and the potential for PF to add to their workload.

 Our findings align closely with a recent evaluation of the Community Pharmacy Contractual Framework in England which found that while staff were motivated to deliver clinical services, they were hampered by financial pressures, inadequate service remuneration, and infrastructure challenges. Poor IT integration with general practice, limited access to medical records, complex referral pathways and limited awareness of community pharmacy provision, further hindered service delivery.^6^ These barriers to implementing clinical services in community pharmacy are not unique to England, with evidence that they are pervasive across several high income countries.^9,20,21^ The prevalence of similar barriers across successive community pharmacy initiatives raises questions about why the sector is repeatedly positioned as a policy solution to rising demand in general practice. Although community pharmacy’s accessibility and skilled workforce make it an attractive route for rapidly expanding primary care capacity, persistent structural constraints particularly funding, workforce capacity, and IT integration continue to limit the extent to which learning from previous schemes is embedded in new policy design, undermining the conditions needed for these initiatives to achieve their goals.

 A key barrier to PF implementation was inadequate communication and information sharing, leading to misunderstanding of the policy, particularly among GPs. General practice was not well informed about PF and together with a lack of robust information systems and poor communication between community pharmacy and general practice, day to day PF implementation was hampered. The rapid rollout of all PF seven pathways at once was compounded by ‘policy layering’ whereby new policies are implemented without the removal of pre-existing ones. PF was implemented on top of pre-existing clinical services such as minor ailments schemes, CPCS and locally commissioned services all of which treat signs and symptoms that overlap with those associated with the seven PF conditions.^22^ Policy layering^23,24^ involves adding policies to address new but potentially competing policy problems, with similar or contradictory goals that are less mutually supportive. This contrasts with similar community pharmacy schemes in Scotland and Wales that seemingly adopted ‘smart layering’ or incrementalism^25,26^ gradually adding services over time. Scotland’s integration of the Minor Ailments Service into PF, used by around a third of the public between October 2022 and September 2023, illustrates the benefits of incrementalism.^27^

 Our findings show that relationships between health professional groups can influence policy implementation. Owen-Boukra et al,^28^ identified key factors necessary to support integrated working between community pharmacy and general practice in England. These include: pharmacists should complement, not replace GPs; patients trust services more when sectors work collaboratively; funding models must be supportive and do not perpetuate competition; new policies should be well-planned and resourced, ensuring that patients do not fall between service gaps. These findings offer important lessons for PF policy, particularly in addressing ongoing concerns from the GP perspective, such as; frustration among GPs about the potential of PF to increase their workload, limited inter-operability of information systems, understanding of clinical criteria of PF pathways and concerns about patient safety. These issues reflect wider GP frustration over reduced real-terms funding, investment into non-GP schemes, and a sense of being undermined by government, a sentiment heightened by the acrimonious debate over Physician Associates.^29-32^

 A key impediment to implementation of PF is a lack of interoperability of community pharmacy and general practice information systems. Inadequate IT infrastructure adds to the administrative burden in both sectors, contributing to the growing volume of “hidden work.” GPs undertake substantial non-patient facing work characterised by interpreting test results, drafting referrals and accepting interruptions from clinical colleagues.^33^ Despite such work being associated with patient care it exerts an additional burden and is largely unremunerated. “Hidden work” is not yet documented in community pharmacy, but we posit that PF and other clinical services will inevitably create additional tasks for pharmacists and information system issues are likely to compound existing workloads. For example, pharmacies have to spend time with a patient before it can be established whether they are eligible for one of the PF pathways – this work is unpaid.

 The implementation of PF brings potential unintended consequences. PF is one of several clinical services introduced in community pharmacy in recent years, but it is arguably the most high-profile and demands the greatest shift in workforce capacity and working routines. While the community pharmacy sector has welcomed the opportunity to deliver PF, it has also increased its workload. The 2023 Community Pharmacy England workforce survey reported an 18% pharmacist vacancy rate and a 27% vacancy rate for accuracy checkers.^34^ The cost of hiring additional staff is also likely to be prohibitive. Moreover, PF was preceded by the Additional Roles Reimbursement Scheme^35^ which has funded general practice to expand its workforce to include non-medical healthcare professionals as a means to alleviate burden on GPs and hence increase access to primary care. The scheme has recruited pharmacists to support with management of long-term conditions, conduct medication reviews, and advise on medication effectiveness and safety. Our findings suggest that this scheme may risk compounding existing workforce shortages in community pharmacy. Recent policy changes aimed at reducing pharmacists’ dispensing role such as legislation permitting pharmacy technicians to supply under PGDs and proposed shifts toward remote supervision may ease pressures on pharmacists, allowing them to focus on clinical services.^36,37^

 PF may also disrupt long-standing dispensing-focused workflows in community pharmacy, as consultation can take up to 30 minutes plus documentation time.^38^ This can delay dispensing of medicines and hence affect patient experience. It may also prompt a move toward structured systems like appointments for PF (as highlighted in this study), improving access in comparison to general practice but reducing PF’s accessibility and the walk-in nature of care. As community pharmacies embrace more clinical roles amid rising Independent Pharmacist Prescriber numbers and national policy shifts, PF could drive lasting changes in staffing and routines.

 Variable uptake of PF due to low public awareness and limited GP referrals has resulted in some community pharmacies missing activity thresholds for the £1000 monthly payment,^39^ despite monthly targets being adjusted to a lower level. This has affected community pharmacy engagement and morale. In response, NHS England increased consultation fees and tiered thresholds in June 2025 and more recently reduced payment thresholds (gateway) across the seven clinical pathways, reducing the likelihood of PF consultations being unremunerated. Incentive structures are critical, as dispensing fees decline, clinical services increasingly represent a vital income stream for community pharmacies in England. This contrasts with international trends where dispensing-focused funding models discourage clinical service adoption.^40^

 This study provides important lessons for other countries wishing to implement similar models to PF. Firstly, clear, tailored communication is essential to raise awareness among several stakeholder groups including GPs (and their staff) and the public, build understanding, and drive engagement especially for high-profile national initiatives like PF. Despite standardised protocols, implementation will vary due to the diverse nature of community pharmacies. Policy-makers should also provide clearer guidance to the public and GP teams on which symptoms PF covers and how it integrates with other pharmacy services. Secondly, robust information infrastructure to reducing administrative burden caused by pharmacies’ limited access to patient GP records is key. Thirdly, policy-makers should identify and replicate effective collaboration models between community pharmacies and general practices to support PF delivery and uptake. Strong partnerships enhance service quality and patient outcomes, making collaboration a cornerstone of PF and similar services.^28^ Finally, large-scale policies like PF must include workforce impact analysis. For community pharmacy, this should inform a broader workforce strategy to support expanding services and attract and retain future pharmacists, especially the 2026 cohort of pharmacists who will graduate with Independent Prescriber qualifications.

###  Strengths and Limitations 

 This study combines two commonly used frameworks from policy analysis and implementation science to bridge a gap in evaluating policy across pre-, during-, and post-implementation phases. Applied to a high-profile national policy, the integrated approach addresses key limitations of each framework. While the CFIR has been critiqued for its limited utility in early policy stages and weak links to policy outcomes, the policy triangle helps fill this gap.^41^ Conversely, the policy triangle lacks detailed guidance on contextual analysis an area where CFIR adds value.^42^ Many of the results are structured using the CFIR, hence future use of the integrated framework would require further and deeper analysis of the data using the Policy Triangle. Although further evaluation is needed, this integrated framework shows promise for assessing large-scale national policy implementation.

 The interview period covers the first 4-10 months of the service only and the subset of interviewees from the frontline (CPs and GPs) was small. However, we interviewed a diverse sample of stakeholders at the national level across England and hence, we have been able to develop a comprehensive understanding of PF from the perspective of policy and decision-makers and national sector representatives from community pharmacy and general practice.

## Conclusion

 The implementation of PF in England reflects both considerable policy ambition and the complexities of shifting tasks from GPs to other health professionals. While broadly welcomed for its potential to improve access and reduce pressure on general practice and expand the clinical role of a pharmacist, PF has faced structural, financial, and communication challenges to date. It has added strain to an already stretched workforce, disrupted workflows, and highlighted gaps in collaboration and IT infrastructure. Variable uptake, workforce shortages, and a preference toward more financially viable clinical services in community pharmacy risk undermining its long-term impact. Nonetheless, PF presents an opportunity to reposition community pharmacy within primary care, contingent on clear communication, workforce and integrated IT infrastructure planning, sustainable funding, and cross-sector engagement. Its rollout offers important lessons for future policy development in community pharmacy and primary care more broadly, both in England and beyond.

## Acknowledgments

 The authors would like to acknowledge external colleagues who supported with identifying appropriate interviewees in the first instance as well as the research participants for giving up their valuable time to participate in this study.

## Disclosure of artificial intelligence (AI) use

 Not applicable.

## Ethical issues

 This study received ethical approval from the LSHTM research ethics committee (Reference number: 30430). Researchers approached interview participants by email outlining the purpose of the study and the interview process. Written informed consent was obtained from each participant prior to interview. Participants were assured of confidentiality and anonymity and that participation was voluntary, and that they were free to withdraw from the study. No participants withdrew their consent.

## Conflicts of interest

 Authors declare that they have no conflicts of interest.

## Supplementary files


Supplementary file 1 contains the topic guide.
