# Supplementary material for: Understanding the Factors Involved in the Development and Early Implementation of "Pharmacy First" Services for the Management of Common Conditions in England
Source: Int J Health Policy Manag. 2026 May 24;15:9442. doi: 10.34172/ijhpm.9442 (PMC13498984; doi:10.34172/ijhpm.9442)
Supplement: Supplementary file 1 [file ijhpm-15-9442-s001.pdf]

Article title: Understanding the Factors Involved in the Development and Early Implementation of "Pharmacy First" Services for the Management of Common Conditions in England

Journal name: International Journal of Health Policy and Management (IJHPM)

Authors' information: Mirza Lalani[1]*, Isaac Chu[1], Agata Pacho[1], Ayodeji Matuluko[1], Basharat Hussain[2], Claire Anderson[3], Nicholas Mays[1], Rebecca E. Glover[1,4]

[1]Department of Health Services Research and Policy, London School of Hygiene & Tropical Medicine, London, UK.

[2]School of Medicine, University of Nottingham, Nottingham, UK.

[3]School of Pharmacy, University of Nottingham, Nottingham, UK.

[4]Department of Public Health, Environments and Society, London School of Hygiene & Tropical Medicine, London, UK.

***Correspondence to:** Mirza Lalani; Email: mirza.lalani@lshtm.ac.uk

Citation: Lalani M, Chu I, Pacho A, et al. Understanding the factors involved in the development and early implementation of "Pharmacy First" services for the management of common conditions in England. Int J Health Policy Manag. 2026;15:9442.doi:10.34172/ijhpm.9442

Supplementary file 1

Topic guide:

Preliminary questions

What is your current role?

How have you been involved with the conception, development or implementation of Pharmacy First?

Goals and intentions for PF

Tell me about the reasons for developing PF in the first place?

Why was it decided to implement the PF service now?

What are the key goals and expectations for the service?

What hopes or concerns do you have about implementing the service?

What is the main change PF is intended to produce?

Designing and developing PF

How were the seven conditions chosen?

- Were there any disagreements about deciding which conditions to choose? If so, which conditions in particular?
- In your opinion, were any conditions prioritised/ranked more highly?
- Were any other conditions considered? If so, why were they not part of the service?

Why was it decided to introduce all seven conditions at the same time?

Why was deferred antibiotic use not considered?

What other options were considered (as alternatives to PF) that may have had the same impact in terms of meeting the overall goals of PF?

What are the key advantages and disadvantages of PF over previous service provision?

What kind of information or evidence are you aware of that shows whether or not PF will work as intended?

What specific evidence was drawn upon when developing the service?

- Previous similar schemes such as Minor Ailments or CPCS and other local pilots in England
- The devolved nations' pilots and rollout of wider schemes that share features with PF?

What other aspects of the analysis of the evidence for PF and arguments advocating its potential, were presented to convince Ministers about the service?

Implementation of PF

Who were the key influential individuals to get on board with this implementation?

- What are these individuals saying about PF?

To what extent will they influence others' use of the PF service?

In your opinion how do you think the implementation of PF is going?

- Tell me about any early issues you have identified?

What have you noticed about the engagement of community pharmacy with PF?

- How does it differ across the different constituencies and characteristics across the sector;
    - Multiples v independents
    - Geography
    - Locums

What steps were taken to encourage community pharmacists to commit to using PF?

What costs have been incurred in designing and implementing PF?

Are there any ongoing implementation costs now that PF is launched?

Does the real cost (in terms of time for example) of actually providing PF vary between different pharmacies?

To what extent are the payments for the PF commensurate with service provision?

To what extent did you consult other stakeholders of PF, such as GPs/other HCP professionals or leaders, when developing and implementing the service?

How have these stakeholders responded to the PF service?

How will the (infra)structure of a community pharmacy affect the implementation of PF?

- Multiples vs independents
- Changes to pharmacy layout (building a consultation room, impingement on retail space)
- Staffing e.g. locums

What kinds of other structural changes are community pharmacies making to accommodate the PF services?

How would you describe the approach to informing community pharmacy about PF?

What are your thoughts on the training provision provided to community pharmacy about PF?

To what extent was the sector prepared to implement PF?

- What, if anything, would you have done differently to support the readiness of the sector to implement PF?
- How do you think community pharmacy issues such as closures, reduced funding and medicines shortages will affect implementation?

Impact and implications of PF

To what extent are policymakers/sector leaders/pharmacy profession/General Practice in favour (or not) of PF?

- Why do you think is this the case?

Broadly, in your opinion, how have patients responded to the PF service?

Which patient groups are expected to benefit most from PF?

Which patient groups may find it difficult to benefit from PF?

To what extent do you think PF will meet the needs of community pharmacy and patients?

- Improved access to services?
- Impact on General Practice i.e. more time for GPs to address complex cases?
- Quicker diagnosis?
- Help with self-management?
- Reduced travel time and expense?
- Improving relationships between CP and GPs

Which of these is the most important?

Which of these is most likely to happen?

How well does PF fit with the values and norms of community pharmacy?

- Desire among pharmacists to adopt more clinical work?

To what extent did you take into account the potential impact on other core pharmacy services as a result of PF?

How well does PF fit with existing work processes and practices in community pharmacy?

- Have these changed to accommodate PF?
- What are likely issues or complications that may arise?

How does PF affect the private services offered by community pharmacies e.g. travel advice, flu immunisations etc.

Before we finish, I want to take you back to the goals of PF discussed earlier.

- How are you monitoring these goals for progress?
- Do you receive any feedback reports about the PF service? If so, tell us about these reports; type of information, how useful or actionable is the information?
- How do you intend to use this information?

Is there anything in particular that you think the evaluation should be keeping an eye on, especially something that might be overlooked?

Was there anything else you were expecting to discuss that we may have not during the course of the interview?

Do you have any questions for us?
